# Respiratory surveillance wards as a strategy to reduce nosocomial transmission of COVID-19 through early detection: The experience of a tertiary-care hospital in Singapore

**DOI:** 10.1017/ice.2020.207

**Published:** 2020-05-08

**Authors:** Liang En Wee, Jenny Yi Chen Hsieh, Ghee Chee Phua, Yuyang Tan, Edwin Philip Conceicao, Limin Wijaya, Thuan Tong Tan, Ban Hock Tan

**Affiliations:** 1 Singhealth Infectious Diseases Residency, Singapore; 2 Department of Infectious Diseases, Singapore General Hospital, Singapore; 3 Department of Internal Medicine, Singapore General Hospital, Singapore; 4 Department of Respiratory and Critical Care Medicine, Singapore General Hospital, Singapore; 5 Department of Infection Prevention and Epidemiology, Singapore General Hospital, Singapore

## Abstract

**Objectives:**

Patients with COVID-19 may present with respiratory syndromes indistinguishable from those caused by common viruses. Early isolation and containment is challenging. Although screening all patients with respiratory symptoms for COVID-19 has been recommended, the practicality of such an effort has yet to be assessed.

**Methods:**

Over a 6-week period during a SARS-CoV-2 outbreak, our institution introduced a “respiratory surveillance ward” (RSW) to segregate all patients with respiratory symptoms in designated areas, where appropriate personal protective equipment (PPE) could be utilized until SARS-CoV-2 testing was done. Patients could be transferred when SARS-CoV-2 tests were negative on 2 consecutive occasions, 24 hours apart.

**Results:**

Over the study period, 1,178 patients were admitted to the RSWs. The mean length-of-stay (LOS) was 1.89 days (SD, 1.23). Among confirmed cases of pneumonia admitted to the RSW, 5 of 310 patients (1.61%) tested positive for SARS-CoV-2. This finding was comparable to the pickup rate from our isolation ward. In total, 126 HCWs were potentially exposed to these cases; however, only 3 (2.38%) required quarantine because most used appropriate PPE. In addition, 13 inpatients overlapped with the index cases during their stay in the RSW; of these 13 exposed inpatients, 1 patient subsequently developed COVID-19 after exposure. No patient–HCW transmission was detected despite intensive surveillance.

**Conclusions:**

Our institution successfully utilized the strategy of an RSW over a 6-week period to contain a cluster of COVID-19 cases and to prevent patient–HCW transmission. However, this method was resource-intensive in terms of testing and bed capacity.

In the COVID-19 pandemic caused by the novel coronavirus, SARS CoV-2, attempts at containment have the best chance of reducing mortality.^1^ As part of containment efforts, heightened surveillance is necessary to prevent sustained transmission in new locations.^2^ Early isolation of patients with probable or suspected COVID-19 is important to reduce the likelihood of nosocomial spread of the disease. Early reports highlighted significant nosocomial transmission, with almost one-third of patients comprising healthcare workers (HCWs) and hospitalized inpatients.^3^ However, patients with COVID-19 may present with respiratory syndromes indistinguishable from those caused by common respiratory viruses,^4^ which poses a challenge for early isolation and containment.

During previous outbreaks of respiratory disease caused by novel pathogens, such as severe acute respiratory syndrome (SARS) and Middle Eastern respiratory syndrome (MERS), various admission strategies were utilized for containment, such as isolating all patients with febrile pneumonia^5,6^ or a history of travel to at-risk areas.^7,8^ However, fever may not occur in all patients with COVID-19 on initial presentation.^9^ With significant community transmission, the value of a travel history invariably declines. Given the devastating consequences of onward nosocomial transmission,^10^ screening all patients presenting with respiratory syndromes for COVID-19 has been advocated as a strategy.^11^ However, the practicality of such a resource-intensive effort has yet to be studied.

In Singapore, the first imported case of COVID-19 was reported at the end of January 2020; followed by the first documented case of local transmission in early February 2020.^12^ By the end of February 2020, most cases were locally transmitted.^13,14^ A substantial proportion of cases were detected by enhanced surveillance, in which patients who did not fulfill case criteria for COVID-19 were selected for testing.^15^ Here, we report our experience with a novel concept, a respiratory surveillance ward (RSW), which was introduced as a strategy for admission, triage and disposition of patients presenting with respiratory syndromes during a SARS-CoV-2 outbreak.

## Methods

### Institutional setting and study period

Singapore General Hospital (SGH) is a 1,785-bed, public, tertiary-care hospital in Singapore. On average, almost 2,000 cases of pneumonia are admitted through the emergency department (ED) each year, or ~36 patients per week.^16^ At our hospital, most patients are nursed in multibed cohort rooms rather than in single-occupancy rooms. We describe the experience with our institution’s RSWs over a 6-week period from February 5 through March 18, 2020, during the SARS-CoV-2 outbreak with community transmission.

### Respiratory surveillance wards (RSWs): Admissions criteria, layout, infection control, and transfer criteria

At our institution, high-risk patients that fulfilled suspect case criteria for COVID-19 were admitted to an isolation ward with 37 negative-pressure rooms. For protection, staff in the isolation ward used N95 masks, eye protection (face shields), and disposable caps, gowns, and gloves. In general, the official suspect case criteria from our local Ministry of Health comprised a compatible clinical syndrome (pneumonia or acute respiratory disease of varying severity), together with a history of travel to high-risk countries affected by COVID-19, and/or epidemiologic risk factors (eg, contact with a confirmed case of COVID-19).^17^ Our institution employed a broader set of internal screening criteria in our triage process in the emergency department (ED) to identify and isolate suspected COVID-19 cases early, with a cumulative sensitivity of 84.3% over the first 3 months of the outbreak.^17^

However, given the presence of ongoing local transmission, we recognized that patients presenting with respiratory syndromes, but without any suspicious travel history or epidemiology links, might still have unsuspected COVID-19. Hence, all admissions with concomitant respiratory syndromes, without a travel history in the past 14 days or epidemiologic risk factors, were first admitted to the RSWs where COVID-19 was ruled out. At the point of admission to the RSW, a distinction was made between pneumonia (defined as the presence of infiltrates on the chest radiograph) and upper respiratory tract infection (URTI, defined as the presence of respiratory symptoms such as breathlessness, cough, coryzal symptoms, but with a normal chest radiograph). For inpatients who did not have respiratory syndromes on admission but subsequently developed symptoms within 14 days of admission, primary physicians could discuss the possibility of transfer to the RSW with an infectious diseases (ID) physician.

Over the study period, 115 beds were set aside for the RSWs, comprising 38 single rooms (with dedicated bathroom) and 77 beds in cohort rooms (with 2–3 patients to a room and shared bathrooms). This ward comprised almost 10% of our hospital’s bed capacity. Single rooms or cohort rooms without any other patients were prioritized for admissions prior to the utilization of shared cohort rooms. Patients suspected of having viral pneumonia (eg, normal procalcitonin, lymphopenia) were also prioritized for nursing in single rooms, depending on the clinical judgement of the attending physician and bed availability. RSWs were run by respiratory medicine or internal medicine specialists. Smaller subspecialty cohorts were created for oncology, cardiology, renal and surgical inpatients; these cohorts were run by designated clinical leads from these subspecialties. In the RSW, we recognized the small potential risk of an unsuspected COVID-19 case; thus, a risk-stratified approach was employed for the use of personal protective equipment (PPE). At the onset, N95 masks were used when handling patients with pneumonia, and surgical masks were used for handling patients with URTI alone. From February 28 onward, given a rising number of unlinked cases detected in the community, N95 masks were used throughout the RSWs. From March 18 onward, given the increased case detection in our RSW, healthcare workers (HCWs) used full PPE including N95 masks, disposable gowns, gloves, and face shields. Within the RSW, social distancing measures were employed; patients were advised to avoid mingling and provided surgical masks to wear at all times, and no visitors were allowed. Beds were spaced at least ∼2 m apart by reducing the number of beds in a cohort room from 6 to 3, and partitions were placed between patient beds.^18^ Patients admitted to the RSW could be transferred for clinically urgent procedures/imaging investigations prior to the results of SARS-CoV-2 tests. In this case, patients wore surgical masks when being transferred and HCWs transporting patients wore N95 masks with PPE at the receiving end, adapted to the kind of procedure being performed (eg, full PPE for all potential aerosol-generating procedures). Patients in the RSW had access to full inpatient services, including allied health services, because our objective was to maintain infection control measures but not compromise patient care.

Patients admitted to the RSW for pneumonia had respiratory samples taken for SARS-CoV-2 testing on arrival; patients would only be transferred if SARS-CoV-2 tests were negative on 2 consecutive occasions at least 24 hours apart.^19^ The requirement for 2 tests 24 hours apart was supported by local studies demonstrating increased testing yield due to the possibility of intermittent viral shedding or variations in sampling technique.^20^ For patients admitted into the RSW with a primary nonrespiratory condition who concomitantly had URTI, SARS-CoV-2 testing was performed if the patient was to be transferred from the designated wards, with the caveat that testing should only be done at day 5 of symptoms. This procedure was supported by local studies that obtained good testing yield when sampling patients presenting with a median of 5 days of symptoms.^20^ Additionally, because patients with URTI symptoms were generally more well, this approach conserved testing resources in the initial phase of the outbreak. From March 1 onward, all patients with URTI alone had 1 swab done on admission, followed by the second swab on day 5 of symptom onset.

### Sampling and microbiological investigations

Respiratory samples (oropharyngeal, nasopharyngeal, or sputum) were processed in our hospital’s laboratory. Investigation for SARS-CoV-2 RNA was conducted using in-house qualitative real-time reverse transcription PCR (rRT-PCR) testing. The routine panel for respiratory virus testing included testing for influenza A, influenza B, respiratory syncytial virus (RSV), rhinoviruses, parainfluenza virus types 1–4, human metapneumovirus, coronaviruses, and adenoviruses. All patients with pneumonia also had sputum and blood specimens collected for culture; if they were deemed to have community-acquired pneumonia, urine was also tested for *Streptococcus* and *Legionella* antigens.

### Ethics approval

This descriptive study was on surveillance data and only aggregate data were collected without patient identifiers; thus, ethics approval was not required under our hospital’s institutional review board guidelines.

## Results

### Patient profile and numbers

**Fig. 1.**
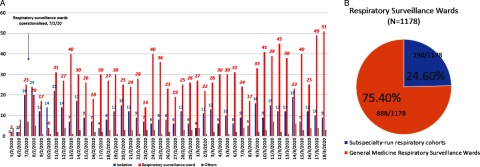
Number of inpatients tested for COVID-19, broken down by isolation wards and respiratory surveillance wards in an acute tertiary hospital in Singapore over a 6-week period during a COVID-19 outbreak.

Over the study period, 1,739 inpatients underwent testing for SARS-CoV-2 in our institution. Of these, 446 (25.6%) were classified as “suspected COVID-19 cases” on admission, given suspicious epidemiological features, and they were directly admitted to the isolation ward. Over the same period, 1,178 patients were admitted to the RSW because they were determined to have symptoms and signs of pneumonia or URTI at the point of ED triage, but they did not have suspicious epidemiological features. The remaining 115 patients were not initially admitted to an isolation ward or the RSW, but they had a SARS-CoV-2 test, either because they were asymptomatic on admission but developed respiratory symptoms within 14 days of admission and approval for testing was given after discussion with an ID physician, or because they were admitted directly to the intensive care unit (ICU). Thus, 1,178 of 1,739 inpatients (67.7%) tested for SARS-CoV-2 came from the RSW (Fig. 1a). In the RSWs, 888 of these 1,178 (75.3%,) were managed within the general medicine RSWs (Fig. 1b); a minority were managed in the subspecialty cohorts.

Among the 888 patients managed in the general medicine RSWs, the mean length-of-stay (LOS) was 1.89 days (SD, 1.23); 319 patients (35.9%) were discharged from the ward and the rest were transferred after their SARS-CoV-2 testing was negative. Over the study period, 5 patients died on the general medicine RSW, but none died from COVID-19. The average age of admitted patients was 68 years (SD, 17.49). Also, 310 patients (34.9%) in the general medicine RSW had a diagnosis of pneumonia confirmed by the managing physician, and 200 (22.5%) had a diagnosis of URTI. The remaining 378 patients had an alternative nonrespiratory or noninfective diagnosis (eg, fluid overload). Differences in demographics, clinical characteristics, and outcomes of the 510 patients with pneumonia or URTI admitted to general medicine RSWs are presented in Table 1. Patients diagnosed with pneumonia, compared with those admitted with URTIs, tended to be older (mean age, 72.4 vs 58.3 years; *P* < .001) and had higher odds of presenting with raised inflammatory markers such as a raised white cell count or procalcitonin. They also had lower odds of being directly discharged from the RSWs (29.0% vs 61.0%; odds ratio [OR], 0.23; 95% confidence interval [CI], 0.17–0.42).

**Table 1.** Differences in Demographics, Clinical Characteristics, and Management Outcomes of Patients Admitted to General Medicine Respiratory Surveillance Wards in an Acute Tertiary Hospital During a COVID-19 Outbreak, Stratified by Pneumonia and Upper Respiratory Tract Illness (URTI)^a^

Variable	Pneumonia (N=310)	URTI (N=200)	Odds Ratio (95% CI)
**Demographics**			
Age, median y (SD)	72.44 (14.96)	58.33 (20.46)	14.10 (10.00–18.20)^†^
Sex, male	166 (53.5)	97 (48.5)	0.81 (0.53–1.27)
**Clinical characteristics**			
Febrile on admission	144 (46.5)	86 (43.0)	0.87 (0.56–1.36)
Leukocytosis on admission	69 (22.2)	22 (11.0)	0.42 (0.22–0.80)^‡^
Raised lactate dehydrogenase	116 (37.4)	74 (37.0)	0.97 (0.62–1.53)
Raised procalcitonin	69 (22.2)	20 (10.0)	0.39 (0.20–0.75)^‡^
**Clinical management outcomes**			
Outcomes			
Discharge	90 (29.0)	122 (61.0)	1.00
Transfer	220 (70.9)^b^	78 (39.0)	0.23 (0.17–0.42)^†^
Delays in care experienced	5 (1.6)	7 (3.5)	2.21 (0.69–7.01)
Length of stay in respiratory ward, mean d (SD)	1.74 (1.03)	1.74 (1.11)	−0.01 (−0.24 to 0.24)

Note. CI, confidence interval; SD, standard deviation.

† *P* < .001.

‡ *P* < .01.

a Means were compared using a *t* test; proportions were compared using a χ^2^ test.

b Includes 5 deaths over the study period.

### Detection of alternative microbiologic diagnosis

**Fig. 2.**
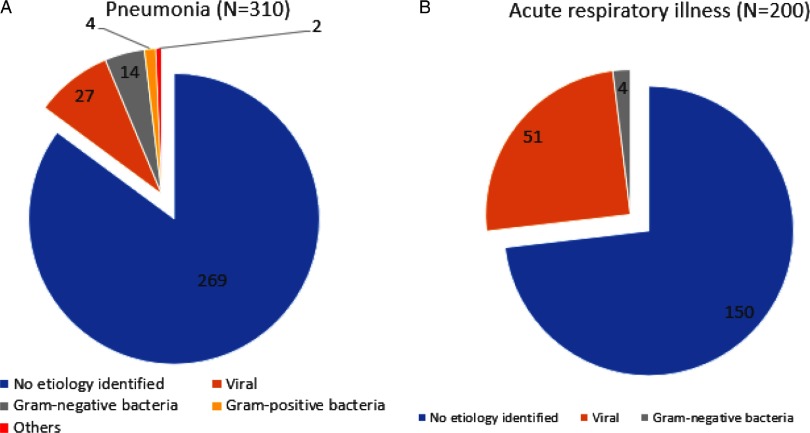
Microbiologic etiology identified for patients with pneumonia/upper respiratory tract illness (URTI) admitted to general medicine respiratory surveillance wards in an acute tertiary hospital during a COVID-19 outbreak.

The consolidated results of patients stratified by pneumonia and URTI are presented in Fig. 2. Among the 310 patients diagnosed with pneumonia, a microbiological diagnosis other than SARS-CoV-2 was obtained for 41 (13.2%) of these patients. In 27 patients, a viral etiology was identified; 18 had a bacterial etiology, 1 patient had pulmonary tuberculosis and 1 patient had *Pneumocystis* pneumonia. The most common viral pathogen identified was rhinovirus (N = 7), followed by parainfluenza (N = 5) and metapneumovirus (N = 3). Among the 200 patients with a URTI, a microbiological diagnosis was obtained for 50 of them (25.0%). In 51 patients, a viral etiology was identified, and 4 had a bacterial etiology. The most common viral pathogen identified was rhinovirus (N = 16), followed by other coronaviruses (N = 8) and parainfluenza (N = 7).

### Case detection

Of the 510 patients with a final diagnosis of pneumonia or URTI admitted to the RSW, 5 (0.98%) tested positive for SARS-CoV-2. Over the same time period, among patients that fulfilled suspect case criteria for COVID-19 admitted to our institution’s isolation ward, 15 of 446 patients (3.36%) tested positive for SARS-CoV-2. Over the same period, no COVID-19 cases were admitted initially to nondesignated areas outside of the RSWs or isolation wards. When cases of COVID-19 were detected, the cases were transferred to the isolation ward, the cohort room was locked down, and any potentially exposed patients (defined as a patient sharing the same cubicle as a confirmed case) were also transferred to the isolation ward as a precautionary measure. In total, 126 HCWs were potentially exposed; however, only 3 of these HCWs (2.38%) required quarantine due to noncompliance with PPE guidelines. No exposed HCWs developed COVID-19 after exposure, despite intensive surveillance over a 14-day period.^21^ HCWs deemed to have significant unprotected exposure based on our local Ministry of Health guidelines were placed under a 14-day quarantine or home isolation, during which they were monitored for respiratory symptoms and submitted temperature measurements twice daily via our institution’s electronic surveillance system. HCWs with contact not amounting to significant unprotected exposure were allowed to continue work but were placed on daily active phone surveillance by our hospital. If symptoms developed within 14 days from the date of exposure, the HCW was instructed to return to the staff clinic for further evaluation and SARS-CoV-2 testing. Among 126 exposed staff, 73 (57.9%) were subsequently tested for SARS-CoV-2 due to the development of symptoms after exposure; all were negative. In total, 13 inpatients were potentially exposed. Of these 13 exposed inpatients, 1 patient subsequently went on to develop COVID-19 after the exposure within the estimated incubation period.^22^ This constituted a cluster of COVID-19 cases with potential nosocomial transmission. In this case, staff had previously observed mingling between the mobile patients in the room, without compliance to surgical masks; subsequently, social distancing was reinforced, and advisories were posted in prominent areas to prevent mingling.^18^

## Discussion

During an outbreak of SARS-CoV-2 with local transmission, an RSW to cohort all inpatients admitted from the community with respiratory symptoms may enhance case detection and reduce the potential of nosocomial transmission.^23^ Despite the apparent low yield of testing in the RSW, our efforts were part of the national strategy of containment of this novel pathogen at that juncture of the outbreak. Our institution drew on experience with SARS, in which exposed patients were triaged, quarantined, and cohorted in open-plan wards,^24^ to conceptualize the RSW. This approach allowed high-risk COVID-19 suspects to be prioritized for management in limited isolation facilities while maintaining vigilance by managing potentially at-risk patients in designated zones to contain the risk of nosocomial transmission.

Aggressive case detection through screening of all patients presenting with acute respiratory infection has been advocated as a potential containment strategy.^11,25^ Our institution employed this strategy over a 6-week period, successfully containing a cluster of COVID-19 infection with potential nosocomial transmission and avoiding patient-HCW transmission amongst exposed staff. Although 5 previously unsuspected cases surfaced in the RSW over the 6-week period, only 13 patients were potentially exposed due to enhanced cohorting, whereas a single patient that surfaced in our institution’s cohorted general ward resulted in the potential exposure of 18 patients.^18^ Given the importance of adequate PPE,^26^ even a single case of COVID-19 can result in the quarantine of large numbers of HCWs if detected late, further straining hospital resources.^27^

However, our study also reflects the practicality and costs of such a resource-intensive effort. Beds set aside for the RSW comprised almost 10% of our institution’s bed capacity. Beds were freed up by reducing elective surgery; however, this method would not be sustainable in the long run. Almost two-fifths of patients entering the RSW were subsequently deemed not to have pneumonia or URTI, despite being originally triaged to these wards from the ED. This was unavoidable as during the outbreak our ED needed to quickly admit patients with suspected respiratory symptoms rather than risk potential exposure in the crowded ED. The requirement for 2 negative tests 24 hours apart reduced bed turnover, reflecting the cost of an aggressive containment strategy. Several strategies might have reduced resource utilization; however, they would have compromised case detection. For instance, reducing the number of negative COVID-19 tests from 2 to 1 would have reduced testing yield^20^; the first COVID-19 case detected in our RSW would have been missed using such an approach. Focusing on febrile patients, as in SARS, was also a consideration.^6^ Only 40% of patients admitted to our RSW had fever (>37.9°C) at presentation to the ED. However, our first case did not present with fever and would have been missed using this strategy.

Our study has several limitations. In our hospital, PCR testing for SARS-CoV-2 was utilized as a diagnostic modality. However, given that the diagnostic yield would likely be dependent on the quality and type of respiratory tract sample, as with other coronaviruses,^28^ COVID-19 cases may have been missed due to sampling issues. Additionally, emerging data suggest the possibility of transmission in presymptomatic patients.^29^ Patients with atypical symptoms and presymptomatic patients could have been missed, similar to our institution’s experience with SARS, in which the index patient in our institution presented in a surgical ward.^30^ Although we did not screen all admissions for COVID-19 due to the logistical challenges involved, 115 patients tested for SARS-CoV-2 did not have symptoms on admission and 378 patients were deemed not to have a diagnosis of pneumonia or URTI on evaluation in the RSW; none of these patients tested positive. A risk-stratified approach of limiting testing to patients with respiratory symptoms on admission, as well as patients who developed respiratory symptoms within a pre-defined incubation period,^22^ might offer a balanced approach to containing COVID-19 during an outbreak with community transmission. In our institution, recognizing the possibility that not all COVID-19 patients may be contained within the isolation ward or RSW, the usage of surgical masks in all clinical areas was made a mandatory minimum for all HCWs. Our results also reflect the experience of a healthcare institution in a COVID-19 outbreak during which the prevailing national strategy was one of containment^13^; exhaustive testing and surveillance may not be feasible in a healthcare system that is overwhelmed.^10^

In conclusion, the strategy of using an RSW was successful in containing patients with COVID-19 in designated areas where enhanced PPE and infrastructural enhancements could potentially reduce nosocomial transmission. A strategy of testing all admitted patients with pneumonia/URTI picked up unsuspected cases of COVID-19, allowing for rapid institution of measures to reduce potential onward transmission.
